# The Influence of an Eight-Week Cycloergometer-Based Cardiac Rehabilitation on Serum Antioxidant Status in Men with Coronary Heart Disease: A Prospective Study

**DOI:** 10.3390/medicina55040111

**Published:** 2019-04-18

**Authors:** Anna Gawron-Skarbek, Jacek Chrzczanowicz, Joanna Kostka, Dariusz Nowak, Wojciech Drygas, Anna Jegier, Tomasz Kostka

**Affiliations:** 1 Department of Hygiene and Health Promotion, Medical University of Lodz, 90-752 Lodz, Poland; 2 Cardiac Rehabilitation Centre, Copernicus Memorial Hospital, Lodz, 90-001 Lodz, Poland; jacekchrz@interia.pl; 3 Department of Physical Medicine, Medical University of Lodz, 90-647 Lodz, Poland; joanna.kostka@umed.lodz.pl; 4 Department of Clinical Physiology, Medical University of Lodz, 92-215 Lodz, Poland; dariusz.nowak@umed.lodz.pl; 5 Department of Preventive Medicine, Medical University of Lodz, 90-752 Lodz, Poland; wojciech.drygas@umed.lodz.pl; 6 Department of Sports Medicine, Medical University of Lodz, 92-213 Lodz, Poland; anna.jegier@umed.lodz.pl; 7 Department of Geriatrics, Medical University of Lodz, 90-993 Lodz, Poland

**Keywords:** exercise training, physical performance, cardiovascular health, exercise physiology, physical activity, antioxidant potential, uric acid

## Abstract

*Background and objectives:* A body of evidence confirms the benefits of cardiac rehabilitation (CR) in coronary heart disease (CHD) patients, but it remains unclear whether it enhances the antioxidant potential. The aim of the study was to assess the influence of an eight-week aerobic cycloergometer-based CR program on serum total antioxidant capacity (TAC) and other CHD risk factors. *Materials and Methods:* The study involved 36 men with CHD (55.2 ± 9.0 years). TAC was assessed with two methods: ferric reducing ability of serum (TAC-FRAS) and 2.2-diphenyl-1-picryl-hydrazyl (TAC-DPPH). Aerobic capacity was evaluated during a submaximal exercise test. TAC and other anthropometric, biochemical and physical activity/fitness measures were performed twice: before the beginning and after termination of CR. *Results:* Aerobic capacity was higher (7.0 ± 2.6 vs. 8.0 ± 2.5 MET—metabolic equivalents; *p* < 0.01), but values of resting diastolic blood pressure were lower (81.9 ± 7.6 vs. 77.4 ± 8.9 mmHg; *p* < 0.01) after termination of CR. Other classic cardiometabolic, anthropometric, and biochemical measures did not change with CR. No difference in TAC-FRAS was found after CR, whereas TAC-DPPH was significantly lower (16.4 ± 4.0 vs. 13.2 ± 3.7% reduction; *p* < 0.01). *Conclusions:* Antioxidant potential measured as TAC-DPPH, but not as TAC-FRAS, decreased with the CR program. The recognized health benefits of CR are not related to augmented serum antioxidant status.

## 1. Introduction

A body of evidence confirms the benefits of cardiac rehabilitation (CR) in patients with coronary heart disease (CHD) [1,2]. A meta-analysis of randomized controlled trials by Taylor et al., [3] assessed the effectiveness of exercise training. Compared to usual care, CR was associated with greater reductions of some CHD risk factors such as total cholesterol, triglycerides and systolic blood pressure (SBP), less significant changes in high- and low-density lipoprotein cholesterol levels and diastolic blood pressure (DBP). The impact of CR on overall mortality was independent of CHD diagnosis, type of CR, dose of physical training, or duration of follow-up [3]. Therefore, regular moderate physical activity is a crucial element of cardiovascular disease therapy, both in ambulatory or in-home conditions.

Some data indicate that physical activity and aerobic capacity (physical fitness) developed during physical exercises may be also important determinants of antioxidant status, both among subjects with CHD and in healthy individuals [4,5,6]. Other studies note a lack of association between physical activity and antioxidant capacities [7,8]. This inconsistency stems from variation in the type of physical efforts (form, frequency, intensity, regularity), the chosen study method and the approach to analyzing the antioxidant compounds (individual antioxidants vs. total antioxidant status; enzymatic vs. non-enzymatic antioxidants), body fluids (plasma, blood serum, saliva), and cells (red blood cells, skeletal muscles). Hence, a number of discrepancies exist between studies, and the present state of knowledge on such dependence is still incomplete [9,10]. Furthermore, it has not been evidenced whether the occurrence of CHD is associated with lower antioxidant potential and whether the CR causes the enhancement of antioxidant capacity. The relationship of CHD with antioxidant defenses may also be modified by many demographic, anthropometric, physiological, and biochemical confounders [11,12,13,14]. Considering these variations, an antioxidant potential assessment should include features of both physical activity and other factors that may affect disease process or potential health benefits.

Total antioxidant capacity (TAC) is assessed by the simultaneous measurement of different elements of the antioxidant defense system. Recently, a promising new method to assess TAC has been developed [15]. It uses the ability of circulating low molecular weight antioxidants to decompose a 2.2-diphenyl-1-picryl-hydrazyl (DPPH) radical to measure their total activity. It was found to exhibit good compliance with the ferric reducing ability of serum (FRAS) [12], a modification of the FRAP method (ferric reducing ability of plasma) [16], mostly by the predominant role played by uric acid (UA) in both tests.

Assuming that TAC may be significantly associated with the recovery process in CHD patients, the aim of the present study was to assess the influence of an eight-week CR program (cycloergometer training, three times per week, 45 min per session) on serum TAC and on other CHD risk factors.

## 2. Materials and Methods

### 2.1. Patients

The study involved 36 men with CHD aged 34.8–73.1 years (55.2 ± 9.0) referred for CR to the Rehabilitation Centre based on medical history, physical examination, and electrocardiogram at rest. Thereafter, they regularly participated in an eight-week CR program in the Cardiovascular and Metabolic Diseases Prevention Centre and Ambulatory Rehabilitation Centre of the Central Clinical Hospital of the Medical University in Lodz (Poland). Aerobic-type exercise training sessions were performed on a cycloergometer (three times per week, 45 min per training unit) with an intensity of 60–80% of heart rate reserve.

Among the CHD subjects, 27 had experienced myocardial infarction (three patients—twice) at least one month earlier, 24 demonstrated arterial hypertension, and 4 had diabetes mellitus. During CR, all the patients received stable drug therapy. An applied pharmacotherapy regimen usually involves aspirin (*n* = 36), statins (*n* = 33), beta-blockers (*n* = 31), angiotensin-converting enzyme (ACE) inhibitors (*n* = 26), ticlopidine (*n* = 24), long-acting nitrates (*n* = 8), diuretics (*n* = 4), calcium channel blockers (*n* = 3), oral antidiabetic drugs (*n* = 3), and fibrates (*n* = 1). All the subjects were free from neoplastic diseases, renal failure, chronic inflammatory diseases, disability, or dementia. Before qualification to the CR program, every patient underwent an individual dietary education (secondary prevention in CHD). Except for cholesterol, glucose, and salt restrictions, none used any special diet. All patients declared that their diet remained unchanged during CR training. The study was approved by the Ethics Committee (RNN/824/2000/KE with KE/733/05 amendment), and all the participants were informed about the aim of the study and gave written informed consent.

### 2.2. Study Protocol and Measures

The study protocol is described in detail in previous cross-sectional studies [12,17]. In brief, the subjects were asked to report for examinations between 8:00–9:00 a.m. after overnight fasting, and restraining from physical exercises, smoking, and alcohol for at least 12 h before laboratory measurements. After fasting blood drawing and a light breakfast, usual smoking and dietary habits were evaluated using the standard questionnaire [18]. Serum TAC and other anthropometric (weight, height, waist and hip circumference, skinfold measurements at four sites: triceps, biceps, subscapula, and supraileum), biochemical (serum total cholesterol—TC, HDL-cholesterol—high density lipoprotein cholesterol, LDL-cholesterol—low density lipoprotein cholesterol, triglycerides—TG, glucose, UA), systolic blood pressure (SBP) and diastolic blood pressure (DBP), habitual leisure time physical activity (LTPA), and aerobic capacity (AC) measures were assessed twice: once before the beginning of CR training (pre-CR training) and once eight weeks later (i.e., after its termination), at least 24 h after an exercise training session (post-CR training).

#### 2.2.1. Anthropometric Data

Body mass index (BMI) (kg∙m^−2^), the percentage of body fat according to Durnin and Womersley [19] and waist-to-hip ratio (WHR) were calculated as previously described [12,17].

#### 2.2.2. Physical Activity Assessment

LTPA was estimated on the basis of the number of hours and minutes earmarked for weekly rehabilitation and recreational/sports activities (kcal∙week^−1^) according to the tables of Fox et al. [20], and the value of energy expenditure was calculated. The subjects were asked to sum up all the rehabilitation and recreational/sports physical activities for one week before blood sample collection.

#### 2.2.3. Aerobic Capacity (Physical Fitness)

Aerobic capacity (AC) (in metabolic equivalents—METs; 1 MET = 3.5 mL·kg of body mass^−1^·min^−1^ of O_2_ consumption at rest) was evaluated during a submaximal exercise test on the Marquette 2000 treadmill with CASE 16 software (GE Healthcare, Milwaukee, WI, USA). The graded exercise test was carried out according to the modified Bruce protocol with every 3 min increments of treadmill speed and inclination in order to achieve at least 85% (75% for men taking beta-adrenergic blockers) of maximal age-predicted heart rate (220–age) with a continuous electrocardiogram tracing or when symptoms imposing the test discontinuation occurred.

#### 2.2.4. Laboratory Measurements

Enzymatic methods using automatic analyzers (Marcel S.A., Zielonka, Poland) were applied to determine serum TC, TG, glucose and UA concentration (CORMAY Liquick Cor-CHOL, Liquick Cor-TG, Liquick Cor-GLUCOSE and Liquick Cor-UA, respectively). HDL-cholesterol was measured by the precipitation method (CORMAY-HDL). LDL-cholesterol was estimated using the Friedewald formula (LDL-cholesterol = TC − (HDL-cholesterol + TG/5) [21].

#### 2.2.5. Total Antioxidant Capacity

Blood samples were incubated for 30 min at 37 °C and then centrifuged for 10 min (4 °C, 1500× *g*) for further TAC measurements. Subsequently the samples were stored at −80 °C for no longer than 30 days prior to the assays of antioxidant activity [15,22]. TAC was assessed with two spectrophotometric methods: FRAS [16] with some modifications [15], and DPPH [15,22]. All individual results were calculated as a mean from three separate measurements. Both tests were performed in parallel using the same laboratory equipment (spectrophotometer (LKB Biochrom Pharmacia, Cambridge, England)) and within the same time frame. TAC-FRAS was expressed in mmol·L^−1^ of formed FeCl_2_ and TAC-DPPH as a percentage of free radical DPPH reduction. Both methods have high reproducibility but they measure selected non-enzymatic (DPPH non-protein) antioxidant capacity in different ways. The precise methodology has been described elsewhere [12,15].

### 2.3. Statistical Analysis

Data were verified for normality of distribution and equality of variances. The paired Student’s *t*-test and non-parametric sign test for pair values were used to compare variables before and after CR. Pearson product moment and Spearman correlations were used to determine the relationships between variables. The potential impact of concomitant diseases and major groups of drugs on pre-CR, post-CR, and changes in TAC and aerobic capacity with CR was assessed with a one-way analysis of variance (ANOVA) and the Mann–Whitney test. TAC-FRAS was normalized using a log transformation and TAC-DPPH using a square root transformation. The results of the quantitative variables were presented as a mean ± standard deviation (SD) and *p* < 0.05 was considered statistically significant for all analyses. The statistical analysis was performed using Statgraphics Plus 5 software (Statgraphics Technologies, Inc, the Plains, VA, USA).

## 3. Results

Hypercholesterolemia (total cholesterol over 190 mg·dL^−1^) was found in nine (25%) subjects, overweight (BMI between 25.0–29.9 kg·m^−2^) and obesity (BMI ≥ 30.0 kg·m^−2^) were diagnosed respectively in 21 (58%) and in 11 (31%) subjects, and visceral obesity (WHR ≥1.0) in 22 (61%) patients. SBP values over 140 mmHg were diagnosed in nine (25%) and DBP over 90 mmHg in 11 (31%) subjects. Almost the whole group (*n* = 35) reported having a history of smoking, but during pre-CR training the majority (92%) was free from the addiction.

**Figure 1:**
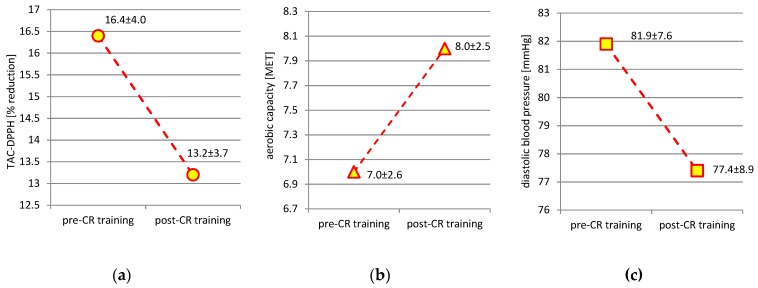
Differences in total antioxidant capacity 2.2-diphenyl-1-picryl-hydrazyl (TAC-DPPH) (**a**), aerobic capacity (**b**), and diastolic blood pressure (**c**) in coronary heart disease patients at the beginning (pre-CR training) and at the end (post-CR training) of the cardiac rehabilitation (CR) program.

Table 1 shows the characteristics of the studied group before (pre-CR training) and after CR (post-CR training). CHD patients had decreased values of DBP at rest during post-CR training (81.9 ± 7.6 vs. 77.4 ± 8.9 mmHg; *p* < 0.01). LTPA energy expenditure and AC were clearly higher after termination of CR. Other classic cardiometabolic, physiologic, anthropometric, and biochemical measures did not change with CR. No difference in TAC-FRAS values was found between pre-CR and post-CR training, whereas TAC-DPPH was significantly lower during post-CR training (Figure 1).

No correlation was found for TAC-FRAS or TAC-DPPH with LTPA or AC, neither at baseline nor at follow-up, although some regular negative tendency should be noted. At baseline, positive correlations were found between DBP and TAC-FRAS and between DBP and UA. During post-CR training, patients with higher DBP were characterized with increased TAC-FRAS and TAC-DPPH (Table 2).

No correlations were identified between changes (Δ) in TAC (neither for Δ TAC-FRAS nor for Δ TAC-DPPH), or changes in LTPA or in AC.

Age had no association with changes of TAC and AC following rehabilitation, or to the baseline and post-CR training TAC values. Both pre-CR training and post-CR training aerobic capacity were negatively related to age (rho = −0.32; *p* = 0.06 and rho = −0.33; *p* = 0.05, respectively).

No significant difference in changes in antioxidant capacity or aerobic fitness associated with CR was observed between sub-populations with and without comorbidities (i.e., myocardial infarction, arterial hypertension, and diabetes mellitus type 2). No differences in TAC-FRAS, TAC-DPPH, or physical fitness, either baseline values or changes following rehabilitation, were observed between subgroups using the main groups of drugs (aspirin, statins, ACE inhibitors, beta-blockers, calcium channel blockers).

## 4. Discussion

The results of the current work indicate that participation in an eight-week CR aerobic training program caused a decline of antioxidant defense as measured by TAC-DPPH, while no change in antioxidant status was revealed by the FRAS test. CR enhanced physical fitness and moderately improved the overall cardiovascular risk profile.

CR is a crucial element of CHD treatment [23]. It has been proven that CR exercises are beneficial for CHD and post-myocardial infarction patients [24], but it remains unclear whether moderate-intensity endurance training, usually taken up during CR programs, may enhance the physiological antioxidant potential. Available data concerning the influence of physical activity, including CR, and physical fitness on oxidative stress markers and the level of antioxidant status in different health conditions is full of controversies. Some reports show a positive effect of systematic physical activity on antioxidant potential [4,25,26,27], others note a lack of this association [7,8], and finally there are studies which demonstrate adverse effects of physical activity on antioxidant capacities [5,28,29].

In several cross-sectional studies (e.g., in the InCHIANTI and ATTICA trials), serum antioxidants and TAC positively correlated with physical activity and physical performance [26,27]. Similarly, higher physical activity resulted in decreased plasma oxidative stress markers (iso-PGF2α and protein carbonyl concentration) and increased erythrocyte superoxide dismutase (SOD) activity in older subjects [30]. Even short-term (only 2 weeks) CR decreased isoprostanes in the urine of non-smoker patients after acute myocardial infarction [31]. However, earlier studies by Kostka et al., did not show any relationship between TAC and physical activity/fitness status in healthy active elderly women [32] or men [33]. Meanwhile, in a cross-sectional population study by Sharpe et al., a negative relationship of physical fitness to TAC was found in female subjects [29].

Intervention studies have also not given consistent data. Eleutério-Silva et al. [8] found increased SOD activity but no significant change in catalase (CAT) activity after six-week combined aerobic and strength CR training in women with metabolic syndrome. Similarly, interval cycloergometer CR training, five times a week, for a total of 15 training sessions increased TAS, SOD-1, and glutathione peroxidase (GPx) activity in acute coronary syndrome patients, but decreased CAT activity [34]. Some reports indicate that only special types and intensities of exercise training may influence antioxidant potential. Schjerve et al. [35] reported that only strength training (twelve-week program; three times per week; high-intensity leg press, abdominal and back strength training) improves TAC but not aerobic exercise, regardless of its intensity.

One possible explanation for the coexistence of a more favorable overall cardiometabolic risk profile (lower DBP, higher LTPA and AC) with lower TAC after the CR program observed in the present study may be associated with physical activity-induced oxidative stress intensification. A high-intensity single exercise session or intensive exercise training period may lead to increased reactive oxygen species production and transient depletion of antioxidant potential [25,28,36]. Moderate physical effort increases expression of mitochondrial antioxidant enzymes [37]. Therefore, physically active subjects may have better cellular antioxidant protection and do not need to maintain high levels of antioxidant defense in the plasma. It is possible that CHD patients are more vulnerable to exercise training, with a more blunted recovery process, so exercise overloading during CR sessions could lead to greater consumption of antioxidants and lowered TAC. The depletion of antioxidant potential observed in patients may be an effect of sustained oxidative stress related to the presence of cardiometabolic risk factors (overweight/obesity, long-lasting smoking habit, or laboratory lipid indices of metabolic syndrome) [5], less common in apparently healthy subjects [17]. Differently, in the course of many diseases, in particular in their early stages or during aggravation, an antioxidant defense system may respond by increasing its activity [36].

Discrepancies observed in the literature may also be related to diversities of methods to assess TAC. It should be emphasized whether measured TAC is the effect of an acute exercise intervention or a long-term training. At least two methods to determine TAC should be applied to receive more solid results [22]. In the present study, two applied methods may be interpreted in different, but complementing ways [15]. TAC-DPPH determines the decrease in absorbance while TAC-FRAS measures formed ferrous ions by increased absorbance [22,38]. Previous studies indicate that obesity, higher blood pressure, or elevated lipids and UA concentration are related to both higher TAC-DPPH and TAC-FRAS [5,12]. UA is the strongest circulating antioxidant preserving vascular dilatation during oxidative stress [15,39]; it accounts for a majority (60–80%) of plasma TAC [40]. The relationship of DBP to TAC and UA in the present study also suggests that increased UA may protect against oxidative stress.

Several shortcomings of the present study should be acknowledged. This is a prospective study performed in CHD patients qualified for the CR program. Our subjects were volunteers, probably more physically active and more prone to undergo exercise testing than it would be the case of a random sample. Applied exercise training was purely endurance of aerobic type—the results of resistance muscle training might have been different. Neither method of TAC assessment measures all the antioxidants occurring in body fluids; therefore, it is desirable to use at least two different TAC methods to give a complete picture of serum antioxidant capacity. Although both DPPH and FRAS tests measure the TAC of blood serum, they reflect the somewhat different physiological properties related to different individual antioxidants present in the sample [15]. This may be the cause of different changes in TAC values following CR program. Despite the limitations of the TAC methodology, measurement of TAC is regarded by some researchers as a more physiologically representative measure of antioxidant protection as compared to individual antioxidants. Notwithstanding, future studies aimed at assessing the potential changes in serum antioxidant parameters in CHD patients after CR training should examine the antioxidant enzymatic activities of SOD, CAT, and GPx. Understanding the mechanisms responsible for the most favorable exercise training being able to optimize TAC and to reduce potential negative effects of oxidative stress seems to have essential meaning for further studies.

## 5. Conclusions

We conclude that regular eight-week periods of CR training increased AC and decreased DBP in men with CHD. Antioxidant potential measured as TAC-DPPH, but not as TAC-FRAS, decreased with the CR program. Simultaneous determination of TAC, antioxidant enzyme activities, and oxidative stress in the CHD patients before and after the CR program should be performed in future studies.

## Figures and Tables

**Table 1:** Selected anthropometric and biochemical characteristics, blood pressure, physical activity, fitness measures, and total antioxidant capacity in coronary heart disease men before (pre-cardiac rehabilitation (CR) training) and after an eight-week cardiac rehabilitation program (post-CR training).

Variable	Pre-CR Training	Post-CR Training
Body mass index (kg·m^−2^)	27.9 ± 3.3	28.0 ± 3.1
Waist circumference (cm)	103.1 ± 7.5	102.4 ± 1.6
Waist to hip ratio	1.00 ± 0.03	1.00 ± 0.04
Percentage of body fat	24.3 ± 4.6	23.7 ± 4.3
Systolic blood pressure (mmHg)	126.4 ± 12.6	123.1 ± 17.2
Diastolic blood pressure (mmHg)	81.9 ± 7.6	77.4 ± 8.9 †
Heart rate at rest	64.3 ± 10.5	64.3 ± 8.4
Total cholesterol (mg·dL^−1^)	163.3 ± 37.7	165.8 ± 37.5
LDL-cholesterol (mg·dL^−1^)	91.0 ± 33.4	93.2 ± 33.7
HDL-cholesterol (mg·dL^−1^)	42.5 ± 7.4	42.7 ± 7.4
TC/HDL-C ratio	3.9 ± 0.9	4.0 ± 1.1
Triglycerides (mg·dL^−1^)	147.9 ± 73.9	154.1 ± 102.5
Glucose (mg·dL^−1^)	91.5 ± 15.5	87.0 ± 7.8
Uric acid (mg·dL^−1^)	6.19 ± 1.42	6.50 ± 1.60
LTPA (kcal·week^−1^)	58.3 ± 167.3	947.6 ± 731.1 ‡
Aerobic capacity (METs)	7.0 ± 2.6	8.0 ± 2.5 †
TAC-FRAS (mmol FeCl_2_·4H_2_O·L^−1^)	1.28 ± 0.24	1.29 ± 0.22
TAC-DPPH (% reduction)	16.4 ± 4.0	13.2 ± 3.7 †

†—*p* < 0.01; ‡—*p* < 0.001; CR—cardiac rehabilitation; DPPH—2.2-diphenyl-1-picryl-hydrazyl; FRAS—ferric reducing ability of serum; HDL-C—high density lipoprotein cholesterol; LDL-C—low density lipoprotein cholesterol; LTPA—leisure time physical activity; MET—metabolic equivalent; TAC—total antioxidant capacity; TC—total cholesterol.

**Table 2:** Correlation coefficients of total antioxidant capacity measures and uric acid concentration to physical activity and fitness characteristics in coronary heart disease men before (pre-CR training) and after cardiac rehabilitation (post-CR training).

	Pre-CR Training			Post-CR Training		
Variable	TAC-FRAS (mmol FeCl_2_∙L^−1^)	TAC-DPPH (% reduction)	UA (mg∙dL^−1^)	TAC-FRAS (mmol FeCl_2_∙L^−1^)	TAC-DPPH (% reduction)	UA (mg∙dL^−1^)
Energy expenditure for LTPA (kcal·week^−1^)	−0.06	−0.09	−0.24	−0.001	0.07	0.06
Energy expenditure for LTPA (kcal·week^−1^·kg^−1^)	−0.04	−0.09	−0.22	−0.19	−0.008	−0.007
Aerobic capacity (MET)	−0.17	−0.14	−0.30	−0.31	−0.27	−0.22
DBP (mmHg)	0.51 †	0.32	0.49 †	0.37 *	0.40 *	0.08

*—*p* < 0.05, †—*p* ≤ 0.01; CR—cardiac rehabilitation; DBP—diastolic blood pressure; DPPH—2.2-diphenyl-1-picryl-hydrazyl; FRAS—ferric reducing ability of serum; LTPA—leisure time physical activity; TAC—total antioxidant capacity; UA—uric acid.
